# Osteoclastic miR-214 targets TRAF3 to contribute to osteolytic bone metastasis of breast cancer

**DOI:** 10.1038/srep40487

**Published:** 2017-01-10

**Authors:** Jin Liu, Defang Li, Lei Dang, Chao Liang, Baosheng Guo, Cheng Lu, Xiaojuan He, Hilda Y. S. Cheung, Bing He, Biao Liu, Fangfei Li, Jun Lu, Luyao Wang, Atik Badshah Shaikh, Feng Jiang, Changwei Lu, Songlin Peng, Zongkang Zhang, Bao-Ting Zhang, Xiaohua Pan, Lianbo Xiao, Aiping Lu, Ge Zhang

**Affiliations:** 1 Institute for Advancing Translational Medicine in Bone & Joint Diseases, School of Chinese Medicine, Hong Kong Baptist University, Hong Kong SAR, China; 2 Institute of Integrated Bioinfomedicine and Translational Science, School of Chinese Medicine, Hong Kong Baptist University, Hong Kong SAR, China; 3 Institute of Precision Medicine and Innovative Drug Discovery, Hong Kong Baptist University, Hong Kong SAR, China; 4 Shenzhen Lab of Combinatorial Compounds and Targeted Drug Delivery in Institute of Integrated Bioinfomedicine & Translational Science, HKBU Institute of Research and Continuing Education, Shenzhen, China; 5 Shum Yiu Foon Shum Bik Chuen Memorial Centre for Cancer and Inflammation Research, Hong Kong Baptist University, Hong Kong SAR, China; 6 Institute of Basic Research in Clinical Medicine, China Academy of Chinese Medical Sciences, Beijing, China; 7 Shenzhen People’s Hospital, Ji Nan University Second College of Medicine, Shenzhen, China; 8 School of Chinese Medicine, Faculty of Medicine, The Chinese University of Hong Kong, Hong Kong SAR, China; 9 Bao’an Hospital Affiliated to Southern Medical University & Shenzhen 8th People Hospital, Shenzhen, China; 10 Guanghua Integrtive Medicine Hospital/Shanghai University of Traditional Chinese Medicine, Shanghai, China

## Abstract

The role of osteoclastic miRNAs in regulating osteolytic bone metastasis (OBM) of breast cancer is still underexplored. Here, we examined the expression profiles of osteoclastogenic miRNAs in human bone specimens and identified that miR-214-3p was significantly upregulated in breast cancer patients with OBM. Consistently, we found increased miR-214-3p within osteoclasts, which was associated with the elevated bone resorption, during the development of OBM in human breast cancer xenografted nude mice (BCX). Furthermore, genetic ablation of osteoclastic miR-214-3p in nude mice prevent the development of OBM. Conditioned medium from MDA-MB-231 cells dramatically stimulated miR-214-3p expression to promote osteoclast differentiation. Mechanistically, a series of *in vitro* study showed that miR-214-3p directly targeted *Traf3* to promote osteoclast activity and bone-resorbing activity. In addition, osteoclast-specific miR-214-3p knock-in mice showed remarkably increased bone resorption when compared to the littermate controls, which was attenuated after osteoclast-targeted treatment with *Traf3* 3′UTR-containing plasmid. In BCX nude mice, osteoclast-targeted antagomir-214-3p delivery could recover the TRAF3 protein expression and attenuate the development of OBM, respectively. Collectively, inhibition of osteoclastic miR-214-3p may be a potential therapeutic strategy for breast cancer patients with OBM. Meanwhile, the intraosseous TRAF3 could be a promising biomarker for evaluation of the treatment response of antagomir-214-3p.

Osteolytic metastasis is the most common form of bone metastasis in patients with breast cancer^1^. The metastatic cancer cell could cause exaggerated osteoclast formation and excessive bone resorption, leading to osteolytic bone metastasis (OBM)^2^,^3^. It is becoming evident that the interaction between cancer cells and the bone microenvironment are important for the development of OBM. However, our knowledge on the underlying molecular mechanism responsible for the aberrantly elevated osteoclastic bone resorption during the development of OBM is still limited.

MicroRNAs (miRNAs) are ~22-nucleotide noncoding RNAs involved in the post transcriptional regulation of gene expression to coordinate a broad spectrum of biological processes^4^,^5^,^6^,^7^,^8^. Although accumulating evidence demonstrates that miRNAs also participate in regulating osteoclast differentiation and activity^9^,^10^,^11^,^12^,^13^,^14^,^15^, the role of osteoclastic miRNAs in regulating the osteoclastic bone resorption under cancer conditions and its contribution to the development of OBM are largely underexplored. More importantly, there is still a lack of miRNAs directly identified from human bone specimens from breast cancer individuals to contribute to the pathophysiological regulation of OBM.

In the present study, we showed that miR-214-3p was significantly upregulated in bone specimens from breast cancer patients with OBM as well as in osteoclasts from nude mice with human breast cancer xenografts (BCX) during OBM development, which was accompanied by the elevated bone resorption. Loss of miR-214-3p gene within osteoclasts prevented the development of OBM in nude mice with BCX. A series of *in vitro* and *in vivo* mechanistic studies demonstrated that miR-214-3p could directly target *Traf3* to promote osteoclastic bone resorption. Finally, we found that osteoclast-targeted antagomir-214-3p treatment could attenuate OBM in nude mice with BCX. Collectively, our data suggest that inhibition of osteoclastic miR-214-3p may be a potential therapeutic strategy for breast cancer with OBM.

## Results

### High miR-214-3p in osteoclasts correlates with elevated bone resorption during the development of OBM

**Figure 1:**
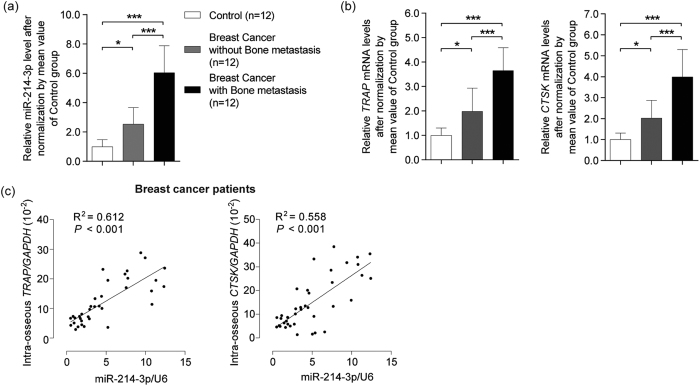
Elevated miR-214-3p level associates with increased bone resorption in bone specimens from breast cancer patients. (**a**) The miR-214-3p level and (**b**) mRNA levels of *TRAP* and *CTSK* in bone specimens from breast cancer patients and cancer-free individuals with fracture (Control). (**c**) The correlation analysis between miR-214-3p level and *TRAP* (or *CTSK*) mRNA level in bone tissue. Note: miR-214-3p levels were normalized to U6 and osteoclast marker genes mRNA levels were normalized to *Gapdh*. *P < 0.05, ***P < 0.001.

We examined the expression profiles of the miRNAs that have been previously reported to promote osteoclastogenesis^16^ and the mRNA expression of bone resorption marker genes (*TRAP* and *CTSK*) in bone specimens from breast cancer patients and cancer-free individuals with fracture (Fig. 1, Supplementary Fig. 1 and Supplementary Table 1). Among the osteoclastogenic miRNAs, the intraosseous miR-214-3p level in OBM patients was significantly higher than that in breast cancer patients without OBM and cancer-free individuals, respectively (Fig. 1a). Meanwhile, the intraosseous mRNA levels of *TRAP* and *CTSK* in OBM patients was also higher than those in breast cancer patients without OBM and cancer-free individuals, respectively (Fig. 1b). Consistently, the serum TRAP-5b levels in OBM patients were significantly higher than those in the relative control groups (Supplementary Table 1). The correlation analysis further revealed that the miR-214-3p level was positively correlated with the mRNA levels of bone resorption marker genes in bone specimens pooled from breast cancer patients with/without OBM (Fig. 1c). These data suggest that the highly expressed intraosseous miR-214-3p were closely associated with the elevated bone resorption in OBM patients. Given that a recent study reported that miR-214-3p targets phosphatase and tensin homolog (PTEN) to promote osteoclastogenesis^17^, we also examined the protein expression of PTEN in the bone specimens. Surprisingly, no obvious differences in the PTEN protein levels were found between the breast cancer patients with or without OBM (Supplementary Fig. 2).

**Figure 2:**
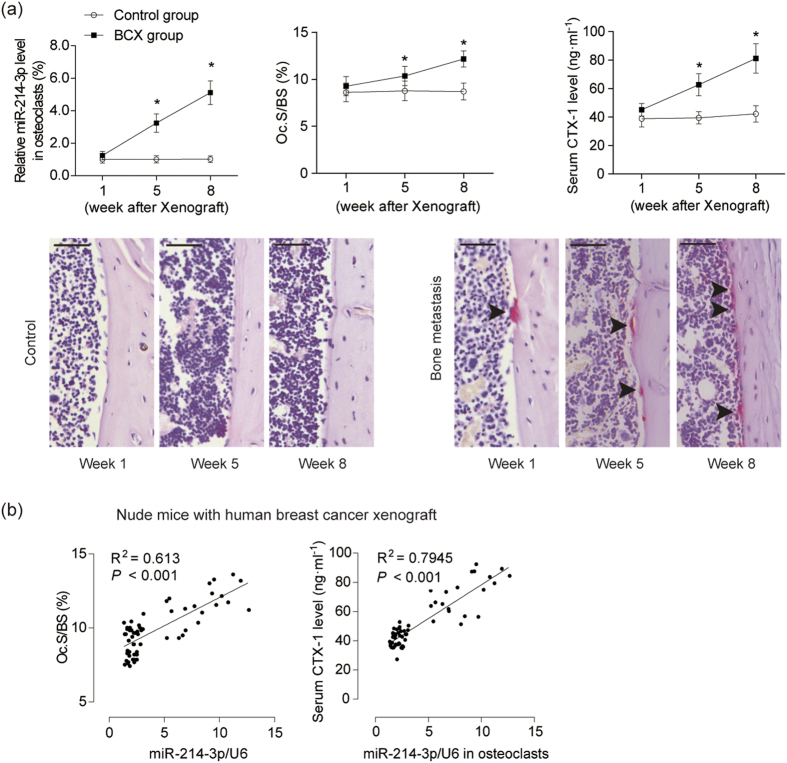
Elevated osteoclastic miR-214-3p level associates with increased bone resorption in bone specimens from BCX nude mice. (**a**) The osteoclastic miR-214-3p levels (upper left), Oc.S/BS in proximal tibia (upper middle), serum CTX-I levels (upper right) and the representative images of TRAP staining in proximal tibia (bottom) were examined (n = 12 for each group). Scale bar: 100 μm. Arrow indicates TRAP-positive cells. The levels of miR-214-3p were normalized to the mean value of control group. (**b**) The correlation analysis between osteoclastic miR-214-3p level and Oc.S/BS and between osteoclastic miR-214-3p level and serum CTX-I levels was performed, respectively. Note: miR-214-3p levels were normalized to U6 and osteoclast marker genes mRNA levels were normalized to *Gapdh*. *P < 0.05 when compared to Week 1 in BCX mice.

Given that miR-214-3p is evolutionarily conserved in human and rodents^18^, we sought to investigate the relationship between osteoclastic miR-214-3p and bone resorption during the development of OBM in mouse models. To mimic the pathological condition of OBM, the nude mice were grafted with human breast cancer MDA-MB-231 cells (hereafter BCX mice) through intraventricular injection and sacrificed at 1, 5 and 8 weeks after xenografts^19^, respectively. In the mouse model, the bone marrow cells positive for osteoclast-associated receptor (OSCAR), a marker specifically expressed in the cell surfaces of pre-osteoclasts and mature osteoclasts^20^, were isolated by magnetic-activated cell sorting (MACS). The mRNA expression of the osteoclast-related marker genes (*Rank, Ctsk* and *Trap5b*) in OSCAR^+^ cells were remarkably higher than those in OSCAR^−^ cells (Supplementary Fig. 3). The osteoclastic miR-214-3p level within OSCAR^+^ cells, the value of Oc.S/BS and the serum bone resorption marker CTX-1 level were all gradually elevated in BCX mice during the development of OBM from week 1 to week 8 after xenografts (Fig. 2a). Moreover, the correlation analysis indicated that the osteoclastic miR-214-3p level was positively correlated with Oc.S/BS and serum CTX-1 levels in this mouse model (Fig. 2b). Collectively, these data imply a close association between the elevated osteoclastic miR-214-3p level and the excessive bone resorption during the development of OBM.

### Loss of miR-214-3p within osteoclasts prevents osteolytic bone metastasis

**Figure 3:**
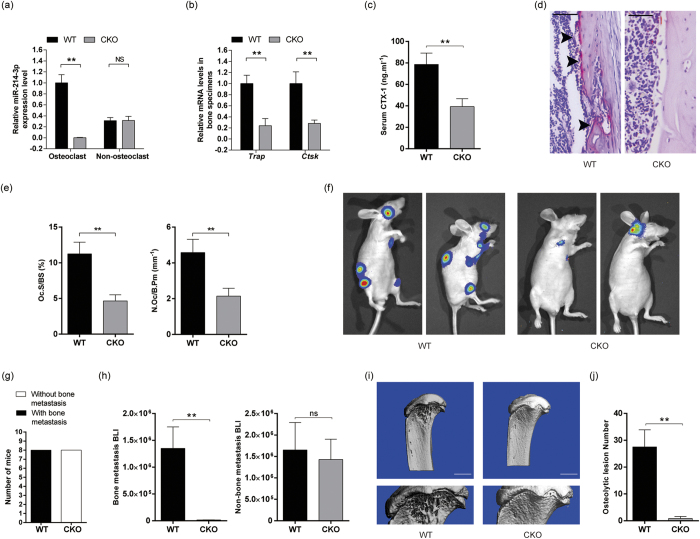
Genetic ablation of osteoclastic *miR-214-3p* prevents osteolytic bone metastasis in BCX nude mice. Q-PCR analysis of the miR-214-3p levels (**a**) and the TRAP and CTSK mRNA levels (**b**) in osteoclasts and non-osteoclasts isolated from bone marrow cells by MACS. (**c**) ELISA assay of serum CTX-I levels. (**d**) The representative images of TRAP staining in distal femur. Scale bar: 100 μm (**e**) Bone histomorphometric analysis for Oc.S/BS and N.Oc/B.Pm in proximal tibia. (**f**) The representative bioluminescence imaging showing the degree of metastasis. (**g**) The number of nude mice with/without bone metastasis in the forelimb, hind limb and spine area detected by bioluminescence imaging. (**h**) The bioluminescence signal intensity at the sites of bone metastasis (left) and non-bone metastasis (right). (**i**) The representative micro-CT images showing the osteolytic bone lesion at distal femur. Scale bar: 1 mm (**j**) The number of osteolytic lesion at distal femur. Note: n = 8 for each group. (**a**,**b**) CKO: osteoclast-specific *miR-214-3p* knockout nude mice, WT: Wild-type nude mice. (**c**–**j**) WT: WT nude mice with BCX, CKO: CKO nude mice with BCX. Data were means ± s.d. **P < 0.01.

To investigate the role of osteoclastic miR-214-3p in the development of OBM, we constructed the heterozygous mice carrying the mutant allele with *LoxP* sites harboring the genomic region of the 106-bp pre-miR as previously reported^21^. Then, the mice were intercrossed with nude mice to obtain the miR-214-3p^*flox*/−^ nude mice, which were then crossed with the *Ctsk-Cre* mice to generate the osteoclast-specific *miR-214-3p* knockout nude mice (Ctsk; *miR-214-3p*^*flox*/*flox*^, hereafter CKO mice) and the relative controls (*miR-214-3p*^*flox*/*flox*^, hereafter WT mice). The miR-214-3p level and the mRNA levels of *Ctsk* and *Trap* were all significantly lower in osteoclasts (OSCAR+ cells isolated from bone marrow by MACS) from CKO mice as compared to WT controls (Fig. 3a,b). To examine the effect of osteoclast-specific *miR-214-3p* depletion on osteolytic bone metastasis, the CKO mice and WT controls were grafted with human breast cancer cells through intraventricular injection and sacrificed at 8 weeks after xenografts. The data showed that CKO mice with BCX had remarkably lower level of serum CTX-1 and lower value of Oc.S/BS and N.Oc/B.Pm as compared to WT controls (Fig. 3c–e). Bone metastasis was markedly diminished in the CKO mice with BCX while no significant difference in the metastasis to the non-bone tissues were found between CKO mice and WT controls with BCX (Fig. 3f–h). In addition, numerous osteolytic bone lesions were detected in WT nude mice with BCX, whereas the osteolytic lesions were hardly detected in the CKO mice with BCX (Fig. 3i,j). Thus, the above findings suggest that loss of *miR-214-3p* within osteoclasts could prevent osteolytic bone metastasis.

### MiR-214-3p directly targets *Traf3* to regulate osteoclast activity *in vitro*

**Figure 4:**
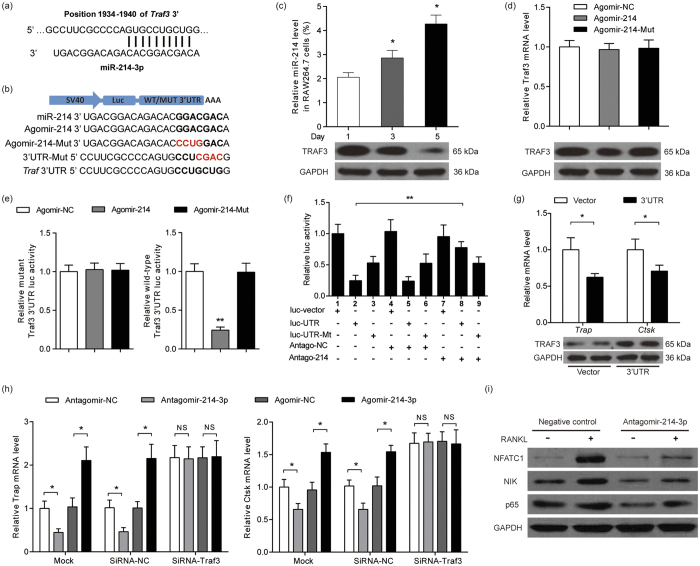
MiR-214-3p targets *Traf3* to functionally promote osteoclast differentiation *in vitro*. (**a**) Sequence alignments between miR-214-3p and candidate binding sites in the 3′UTR of *Traf3*. (**b**) Schematic diagram illustrating the design of luciferase reporters with the WT *Traf3* 3′ UTR (WT 3′ UTR) or the site-directed mutant *Traf3* 3′ UTR (3′ UTR-Mut). The sequences of agomir-214-3p and a agomir-214-3p mutant (agomir-214-Mut) are also shown. Luc, luciferase. (**c**) The relationship between miR-214-3p level (top) and the mount of TRAF3 protein (bottom) during osteoclast differentiation in RAW264.7 cells. (**d**) The effects of agomir-214-3p and agomir-214-Mut on *Traf3* mRNA levels (top) and the amount of TRAF3 protein (bottom) in RAW 264.7 cells. (**e**) The effect of agomir-214-3p and mutated agomir-214-3p on luciferase activity in RAW 264.7 cells transfected with either the WT the mutant *Traf3* 3′ UTR reporter (left) or *Traf3* 3′ UTR reporter (right). (**f**) The effect of antagomir-214-3p on luciferase activity in RAW 264.7 cells transfected with either the WT *Traf3* 3′ UTR reporter or the mutant *Traf3* 3′ UTR reporter. (**g**) Real-time PCR analysis of *Trap* and *Ctsk* mRNA levels (top) and western blot analysis of TRAF3 protein (bottom) in RAW264.7 cells after blockade of miR-214-3p binding to *Traf3* by overexpression of WT *Traf3* 3′UTR (UTR). (**h**) *Trap* and *Ctsk* mRNA levels in RAW264.7 cells after the indicated treatment. The results from a scrambled control siRNA (siRNA-NC) and a mock transfection are also shown. (**i**) Western blot analysis of whole cell lysates of osteoclasts transfected with antagomir-214-3p and cultured with RANKL for 5 days. Note: All data are the mean ± s.d. from three independent experiments. *P < 0.05, NS, not significant.

We next sought to understand how osteoclastic miR-214-3p regulates the osteoclast activity and bone resorption during the development of OBM. The mouse bone marrow-derived macrophages (BMMs) were treated with or without the condition medium (CM) from the cultured MDA-MB-231 cells in the absence of 5 ng/ml RANKL. As expected, the miR-214-3p level and the mRNA levels of *Ctsk* and *Trap* gradually increased during the osteoclast differentiation. Notably, the time-dependent increase in the expression of miR-214-3p, *Ctsk* and *Trap* were more pronounced in the presence of CM from breast cancer (Supplementary Fig. 4a). Further study showed that the mRNA levels of *Trap* and *Ctsk* were upregulated by agomir-214-3p and downregulated by antagomir-214-3p in the RAW264.7 cells treated with RANKL and CM (Supplementary Fig. 4b). Consistently, the number of TRAP-staining positive cells and bone-resorbing activity of osteoclasts was suppressed by antagomiR-214 and promoted by agomir-214 in RAW264.7 cells treated with RANKL and CM (Supplementary Fig. 4c,d). Unexpectedly, no changes in the protein expression of PTEN during the course of osteoclastogenesis (Supplementary Fig. 4e). Given that our data from breast cancer patients also showed that the intraosseous PTEN protein expression seemed to be not affected under pathological conditions of OBM, we next sought to elucidate the mechanisms by which miR-214-3p regulates osteoclastic bone resorption. We used miRBase (version 21) and TargetScan (release version 6.2) to predict the candidate target genes of miR-214-3p, wherein we found that *Traf3* mRNA contains a miR-214-3p binding site in its 3′ untranslated region (3′UTR) (Fig. 4a). A previous study has demonstrated that TRAF3 could limit the RANKL-induced osteoclastogenesis by suppressing canonical and noncanonical NF-κB signaling^22^. Therefore, we sought to investigate the connection between miR-214-3p and TRAF3 during osteoclastogenesis. We observed a gradual increase in the miR-214-3p levels together with a decrease in the amount of TRAF3 protein in RAW 264.7 cells during RANKL-induced osteoclastogenesis (Fig. 4b). Moreover, the protein level but not the mRNA level of TRAF3 was downregulated in osteoclasts treated with agomir-214 treatment, whereas agomir-214-3p mutant containing the mutated binding sites treatment had minimum effect on either the protein or mRNA of TRAF3 (Fig. 4c). To determine whether miR-214-3p could directly target *Traf3*, the RAW264.7 cells were transfected with luciferase reporters containing either a wild-type (WT) *Traf3* 3′UTR or a mutant *Traf3* 3′UTR (Fig. 4d). Agomir-214-3p, but not the agomir-NC and agomir-214-3p mutant, substantially inhibited the luciferase reporter activity of the WT *Traf3* 3′ UTR, whereas the luciferase reporter activity of the mutant *Traf3* 3′ UTR was not repressed by agomir-214-3p (Fig. 4e). Moreover, the luciferase activity of the WT *Traf3* 3′ UTR was notably upregulated after the endogenous levels of miR-214-3p were reduced by antagomir-214-3p treatment (Fig. 4f).

We then transfected the RAW264.7 cells with WT *Traf3* 3′ UTR to block the binding of endogenous miR-214-3p to *Traf3*. The *Trap* and *Ctsk* mRNA levels were both remarkably lower in RAW264.7 cells transfected with the WT *Traf3* 3′ UTR when compared to the cells transfected with vector alone. Accordingly, the level of TRAF3 protein in RAW 264.7 was upregulated after transfection with the *Traf3* 3′ UTR (Fig. 4g). Furthermore, when *Traf3* expression was knocked down by siRNA, the *Trap* and *Ctsk* mRNA consistently remained at a low level under the treatment of agomir-214-3p, antagomir-214-3p and the corresponding negative controls, respectively (Fig. 4h). Moreover, we examined the effect of miR-214-3p on the protein expression level of NFATc1, NIK and p65 (RelA) in RAW 264.7 undergone RANKL-induced osteoclast differentiation. The protein levels of NFATc1, NIK and p65 were all downregulated by antagomir-214-3p when compared to the antagomir-negative control (Fig. 4i). However, no obvious changes in the protein levels of NFATC1, NIK, RelA and RelB after treatment with agomir-214 during osteoclastogenesis when compared to the agomir-negative control (Data not shown).

### Elevated miR-214-3p in osteoclast targets *Traf3* to promote osteoclastic bone resorption *in vivo*

**Figure 5:**
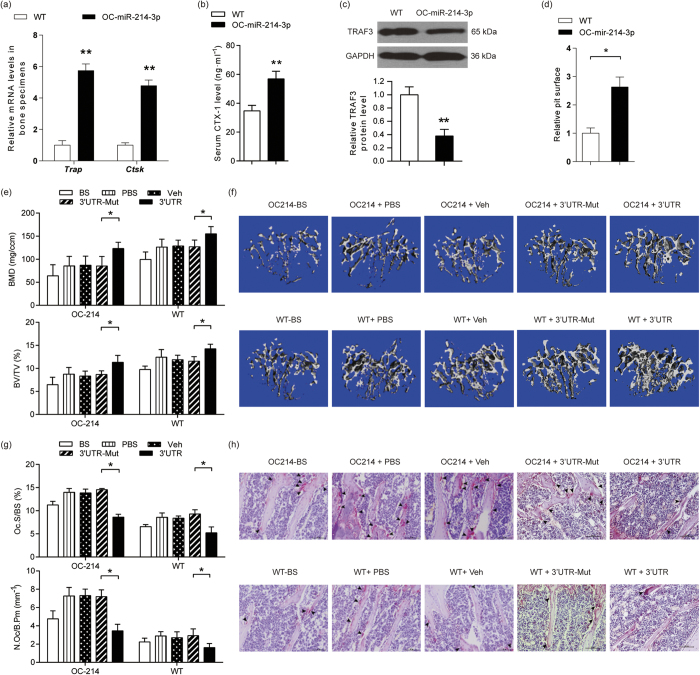
The elevated miR-214-3p within osteoclast targets TRAF3 to promote osteoclastic bone resorption *in vivo*. (**a**) Q-PCR analysis of the mRNA levels of osteoclast marker genes in bone tissue. (**b**) ELISA analysis of serum CTX-1 level from OC-miR-214-3p and WT mice. (**c**) Western blot analysis of the protein expression of TRAF3 in bone tissues. (**d**) *In vitro* bone resorption activity assay showing the bone resorption pit surface of the osteoclasts differentiated from bone marrow macrophages (BMMs) derived from either OC-miR-214-3p mice or WT mice. (**e**) The micro-CT analysis for the BMD and BV/TV of the proximal tibia from the indicated group after treatment. (**f**) The representative micro-CT images for the trabecular micro-architecture in proximal tibia. Scale bar: 1 mm. Note: n = 8~10 for each group. (**g**) Bone histomorphometric analysis for Oc.S/BS and N.Oc/B.Pm in proximal tibia. (**h**) The representative TRAP staining images for the trabecular bone in proximal tibia. Scale bar: 100 μm. Arrow indicates TRAP^+^ cells. Note: n = 6 for each group. Data were means ± s.d. *P < 0.05, **P < 0.01. OC-214: OC-miR-214-3p mice; WT: age-matched littermates; BS: mice that were sacrificed before treatment as baseline; PBS: mice treated with PBS as blank control; Veh: mice treated with (D-Asp)_8_-liposome alone; 3′UTR-Mut: mice treated with (D-Asp)_8_-liposome-mutant *Traf3* 3′UTR plasmid; 3′UTR: mice treated with (D-Asp)_8_-liposome-*Traf3* 3′UTR plasmid.

Next, we investigated the role of osteoclastic miR-214-3p in regulating bone resorption *in vivo* using our previously established osteoclast-specific miR-214-3p knock-in (OC-214) mice that overexpressing miR-214-3p under the control of *Cre*-mediated recombination driven by the *Ctsk* promoter^23^,^24^. Thereafter, we compared the levels of osteoclast activity and bone resorption between the OC-214 mice and WT littermate controls at 8 weeks of age. The mRNA expression of *Trap* and *Ctsk* in bone specimens were remarkably higher in OC-214 mice when compared to those in WT mice (Fig. 5a). The serum CTX-1 level was significantly higher in OC-214 mice than that in WT mice (Fig. 5b). The intraosseous TRAF3 protein levels were notably lower in OC-214 mice when compared to those in WT mice (Fig. 5c). In addition, significantly more pit area formed in the bone slices cultured with the bone marrow-derived osteoclasts from OC-214 mice when compared to those from WT mice (Fig. 5d). All these data suggest that the elevated expression of miR-214-3p within osteoclast could promote osteoclastic bone resorption in OC-214 mice. To investigate whether the elevated miR-214-3p within osteoclasts could promote osteoclastic bone resorption via targeting *Traf3 in vivo*, we evaluated the degree of bone resorption in the OC-214 mice weekly administrated with or without TRAF3 3′UTR-containing plasmid (20 μg per mice) encapsulated in the our previously developed osteoclast-targeted delivery system^25^. The treatment with *Traf3* 3′UTR-containing plasmid but not mutant *Traf3* 3′UTR-containing plasmid could significantly attenuate the increase in bone resorption-related parameters, the decrease in bone mass and the deterioration in trabecular architecture in OC-214 mice (Fig. 5e–h). Collectively, it indicates that the elevated miR-214-3p in osteoclast could target *Traf3* to promote osteoclastic bone resorption *in vivo*.

### Osteoclast-targeted inhibition of miR-214-3p attenuates osteolytic bone metastasis

**Figure 6:**
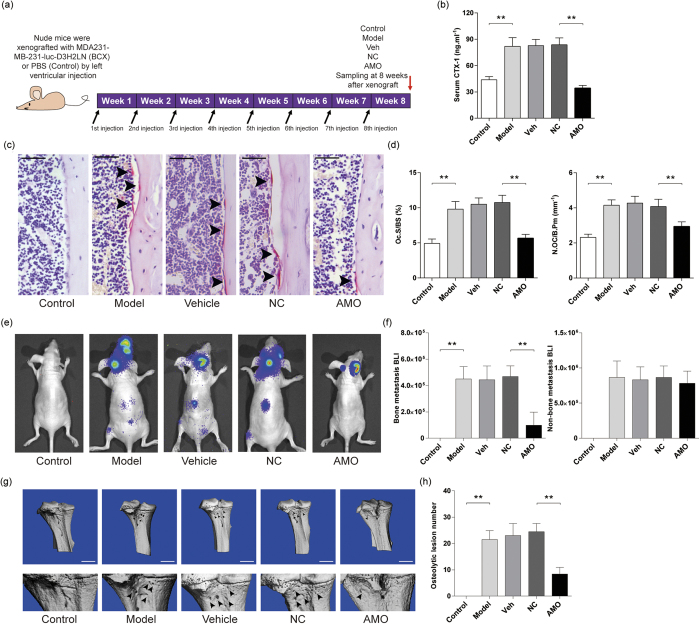
The effects of antagomir-214 delivered by (D-Asp_8_)-liposome on osteolytic bone metastasis in nude mice with breast cancer xenografts. (**a**) A schematic diagram illustrating the experimental design. (**b**) ELISA analysis of serum CTX-1 level in mice from the indicated group after treatment. (**c**) The representative images for TRAP staining in the proximal tibiae bone sections from the indicated group after treatment. Arrow heads indicate osteoclasts. Scale bar: 50 μm. (**d**) The bone histomorphometric analysis for Oc.S/BS and N.Oc/B.Pm in the proximal tibiae bone sections from the indicated group after treatment. (**e**) The representative bioluminescence imaging showing the degree of metastasis in mice from the indicated group after treatment. (**f**) The bioluminescence signal intensity at the sites of bone metastasis (left) and non-bone metastasis (right) in the mice from the indicated group after treatment. (**g**) Representative micro-CT images showing the osteolytic bone lesion at proximal tibiae from the indicated group after treatment. Arrowheads indicate the osteolytic bone lesions. Scale bar = 1 mm. (**h**) The number of osteolytic lesion at proximal tibiae from the indicated group after treatment. Note: n = 12 for each group. The data were presented as the mean ± s.d., **P < 0.01. Control: nude mice without breast cancer xenografts, Model: breast cancer xenografted nude mice (BCX mice) administrated with PBS, Veh: BCX mice administrated with (D-Asp)_8_-liposome, NC: BCX mice administrated with (D-Asp)_8_-liposome-antagomir negative control, AMO: BCX mice administrated with (D-Asp_8_)-liposome-antagomir-214-3p.

We next evaluated the therapeutic effect of inhibiting osteoclastic miR-214-3p on osteolytic bone metastasis. We performed pulsed administration of the antagomir-214-3p encapsulated within osteoclast-targeting delivery system (AMO) in BCX nude mice as described in Fig. 6a. The optimal dosage of antagomir-214-3p was determined in our pilot study, in which almost 80% knockdown efficiency of osteoclastic miR-214-3p was achieved at a dose of 10 mg/kg. In addition, over 50% knockdown was maintained for 8 days after a single dose at 10 mg/kg (Supplementary Fig. 5). Six weeks after the first treatment, the intraosseous miR-214-3p level in BCX nude mice treated with AMO was significantly lower than that in BCX nude mice without AMO treatment (Supplementary Fig. 6a,b), suggesting the efficient inhibition of miR-214-3p in BCX nude mice after AMO treatment. No significant difference in the indexes of liver function (ALT and AST) and kidney function (BUN) were found between the BCX nude mice with or without AMO treatment (Supplementary Table 2). In addition, the intraosseous TRAF3 protein levels in BCX nude mice received AMO treatment were remarkably higher than those in BCX nude mice treated without AMO, whereas the intraosseous PTEN protein levels were not affected by different treatments (Supplementary Fig. 6c). On the other hand, the serum CTX-1 level in BCX nude mice with AMO treatment was also remarkably lower than that in BCX nude mice without AMO treatment (Fig. 6b). Consistently, the values of Oc.S/BS and N.Oc/B.Pm were both significantly lower in those BCX nude mice administrated with AMO when compared to those in BCX nude mice without AMO treatment (Fig. 6c,d). On the other hand, AMO treatment markedly diminished bone metastasis in BCX nude mice (Fig. 6e,f and Supplementary Fig. 6d). In addition, the osteolytic metastatic lesions in tibiae were remarkably alleviated in BCX nude mice after AMO treatment (Fig. 6g,h). These data suggest that therapeutic inhibition of miR-214-3p within osteoclast could attenuates osteolytic bone metastasis.

## Discussion

In this study, we found that elevated miR-214-3p within osteoclast could target TRAF3 to promote osteoclastic bone resorption during the development OBM. Our results suggest that therapeutic inhibition of miR-214-3p in osteoclasts may be a potential therapeutic strategy for OBM with dominant osteoclastic bone resorption.

Previous studies have documented that miRNAs could participate in regulating osteoclastic bone resorption under pathological settings such as osteolytic bone metastasis^19^,^26^. However, these miRNAs were detected from animal studies or *in vitro* experiments. The current study provides the first clinical evidence of a miRNA directly identified from bone specimens of patients with OBM, which contributes to the pathogenesis of OBM. The data from OBM patients as well as murine models concordantly showed a close association between the aberrantly elevated osteoclastic miR-214-3p expression and the increased bone resorption during the progression of OBM. In addition, we have previously shown that miR-214-3p could promote osteoclast differentiation *in vitro*. In this study, we further found that the CM from breast cancer cells could dramatically stimulate the expression of miR-214-3p and induce the osteoclast differentiation *in vitro*, which could be blocked/promoted by antagomir-214-3p/agomir-214-3p treatment. These data suggest that the metastatic breast cancer cells could stimulate miR-214-3p expression in osteoclast precursors to promote osteoclast formation during the development of OBM. On the other hand, it has been proved that miR-214-3p could target p53 to enhance the invasion ability of breast cancer cells^27^, while depletion of miR-214-3p could block the dissemination of breast cancer cells^28^, suggesting an important role of miR-214-3p in mediating breast cancer metastasis. Impressively, we found that osteoclast-specific ablation of *miR-214-3p* could substantially prevent the development of OBM, as evidenced by the low level of CTX-I and bone resorption-related parameters as well as the diminished metastasis to bone in the CKO nude mice after BCX. According to the ‘viscous cycle’ theory^1^,^29^, the interplay between cancer cells and the osteoclasts at the metastasis microenvironment were crucial for the development of OBM. Since we have previously demonstrated that the osteoclast could secrete exosomal miR-214-3p to the bone microenvironment^24^, it was reasonable to expect that the aberrant elevated miR-214-3p levels in osteoclasts may, in turn, contribute to affect the colonized cancer cells at the bone metastatic site.

We further found that miR-214-3p could target TRAF3 to stimulate the osteoclastic bone resorption during the development of OBM. TRAF3 is an important regulator of cytokine production and type I interferons^30^, which is also involved in regulating RANKL-induced osteoclast formation^22^. Consistently, we observed a time-dependent decrease in the TRAF3 protein level, which was accompanied by a gradual increase in the miR-214-3p level, during osteoclastogenesis *in vitro*. In addition, the TRAF3 protein levels in osteoclasts were downregulated after transfection with agomir-214 but not the nonsense agomir control or mutant agomir-214, whereas no differences in the Traf3 mRNA levels were found among different treatments, suggesting a role of miR-214-3p in the posttranscriptional regulation on TRAF3 protein expression. More importantly, the evidences from the luciferase reporter assay and the gain- and loss-of-function experiments indicate that TRAF3 could be a functional target of miR-214-3p. The above *in vitro* findings were further supported by the data from the OC-214 mice showing that osteoclast-targeted treatment with *Traf3* 3′UTR plasmid could remarkably attenuate the excessive bone resorption in OC-214 mice. However, the limitation of this study was that we didn’t examine the effect of miR-214-3p agomir or antagomir on osteoclast differentiation from human peripheral blood mononuclear cells. On the other hand, a recent study shows that miR-214-3p could promote osteoclastogenesis and increase osteoclast activity by targeting PTEN during osteoclast differentiation from bone marrow monocytes (BMMs)^17^. Considering PTEN could regulate bone resorption during the development of OBM, the protein expression of PTEN in the bone specimens was detected. However, we unexpectedly observed little change in the protein expression of PTEN between the breast cancer patients with or without OBM. This could be explained by the previous study showing that the phosphorylated PTEN but not the total PTEN levels decreased during RANKL-induced osteoclast differentiation^31^. Importantly, the BCX nude mice received AMO treatment had a higher TRAF3 protein expression in bone specimens when compared to those without AMO treatment, whereas no obvious changes in the PTEN protein levels were found between the mice with and without AMO treatment. Collectively, it strongly suggests that miR-214-3p could regulate the amount of TRAF3 proteins to contribute to the excessive osteoclastic bone resorption during the development of OBM.

Although controversial, emerging evidence showed that, pharmacological inhibition of osteoclast resorption may increase the survival of patients with OBM^32^. In breast cancer patients, treatment with bisphosphonates decreases skeletal events, such as pathological fracture or bone pain, by 30%~40%^33^. Bisphosphonates also block the formation of bone tumors, and osteoclast inhibition prevents spontaneous osteolytic tumors in HTLV-1 Tax transgenic mice^34^. Thus far, there is no established non-coding RNA target that has been translated into clinical antiresorptive therapy. Considering the distinct role of miR-214-3p described here, we also evaluated the therapeutic effect of inhibiting miR-214-3p within osteoclasts on the development of OBM in BCX nude mice using antagomir-214-3p encapsulated in our established osteoclast-targeting delivery system^25^. The results showed that osteoclast-targeting antagomir-214-3p treatment could dramatically diminish bone metastatic lesions in BCX nude mice, respectively, which could be explained by the decreased bone resorption after knocking down the miR-214-3p level within osteoclasts by antagomir-214. In addition, it has been demonstrated that OBM are always associated with the uncoupled bone formation to bone resorption, *i*.*e*. increased osteoclast activity with reduced osteoblast activity. Given that the osteoclastic miR-214-3p could transfer to osteoblast to inhibit bone formation^24^, the current strategy of inhibiting osteoclastic miR-214-3p may also exert anabolic effect on bone formation. In addition, the intraosseous TRAF3 protein levels was markedly restored after antagomir-214-3p treatment, suggesting that the protein expression of TRAF3 in bone could be a promising biomarker for evaluation of the treatment response of antagomir-214-3p in future translation.

## Materials and Methods

### Bone specimens from patients

We collected the small pieces of dissociated bones from fractured patients during surgery. The postmenopausal women with breast cancer suffering fractures were enrolled. OBM were identified by X-ray or CT imaging. The age-matched postmenopausal women without malignant status were also included. All written informed consent was obtained from each patient or family member before inclusion in this study. All experiments were performed in accordance with relevant guidelines and regulations and all clinical procedures were approved by the Ethics Committees of Shenzhen 8th People Hospital, Shenzhen People’s Hospital and Guanghua Integrtive Medicine Hospital, respectively.

### Agomir-214, antagomir-214 and *Traf3* siRNA

Agomir-214-3p, agomir-negative control (agomir-NC), agomir-214-3p-mutant (agomir-214-Mut), antagomir-214-3p (antago-214) and antagomir-214-3p negative control (antago-NC) were purchased from Guangzhou RiboBio Co., Ltd. The *Traf3* siRNA and negative control (siRNA-NC) were purchased from Invitrogen.

### Mouse Genetic mouse model

The *miR-214-3p *^*flox*/−^ mice were generated as previously reported^21^. Briefly, a mutant allele with *loxP* sites flanking the genomic region encompassing the 106-bp pre-miR and a neomycin resistance cassette were constructed and electroporated into embryonic stem (ES) cells. Positive targeting clones were identified by polymerase chain reaction (PCR) and southern blotting. The targeted ES clones were microinjected into C57BL/6 blastocysts, and male chimeras were mated to C57BL/6 females to obtain *miR-214-3p *^*flox-neo*/+^ mice, which were then intercrossed with *flp*-deleter mice (JAX, 009086) to remove neo cassette in order to generate *miR-214-3p *^*flox*/+^ mice. The female *miR-214-3p* ^*flox*/+^ mice in C57BL/6 background were intercrossed with the male BALB/c nu/nu mice to obtain the *miR-214-3p*^*flox*/+^ nude mice. The female *Ctsk-cre* mice in C57BL/6 background were also intercrossed with the male BALB/c nu/nu mice to generate the *Ctsk-cre* nude mice. Thereafter, the *miR-214-3p *^*flox*/+^ nude mice were crossed with the *Ctsk-cre* nude mice to obtain the *Ctsk;miR-214-3p *^*flox*/*flox*^ (CKO) nude mice and the *miR-214-3p *^*flox*/*flox*^ (wild-type, WT) controls. On the other hand, the OC-miR-214-3p mice were generated according to our published protocols^24^. Firstly, we generate the *Rosa26-PCAG-STOP*^*fl*^-*mmu-miR-214-3p*-knock-in mice. In brief, a cassette containing the following components was constructed to target the *Rosa26* locus: *FRT-LoxP-stop* codons-three SV40 *poly(A*) sequences-*LoxP-mmu-miR-214-3p-WPRE-bGH poly(A)-AttB-PGK* promoter-*FRT-Neo-PGK poly(A)-AttP*. The targeting vector is constructed, fully sequenced and electroporated into C57BL/6 embryonic stem (ES) cells. Positive targeting clones were identified by polymerase chain reaction (PCR) and southern blotting. The targeted ES clones were microinjected into BALB/c blastocysts to obtain chimeric mice following standard procedures. Thereafter, the chimeric mice were intercrossed with C57BL/6 mice to obtain F1 heterozygote mice and then backcrossed with C57BL/6 mice to expand the enough number of heterozygote *Rosa26-PCAG-STOP*^*fl*^-*mmu-miR-214*-knock-in mice. Thereafter, we crossed the *Rosa26-PCAG-STOP*^*fl*^-*mmu-miR-214*-knock-in mice with heterozygous *Ctsk-cre* mice to obtain OC-miR-214 mice. The littermates were used as wild-type control. The *Ctsk*-cre mouse was a generous gift from Prof. Xu Jiake in the West Australia University. It is the offspring of the Ctsk-cre mouse strain established by the Shigeaki Kato’s lab^23^. All experiments were performed in accordance with relevant guidelines and regulations and all experimental procedures were approved by the licensing Committees of Animal Ethics and Experimental Safety of Hong Kong Baptist University.

### Bone resorption activity assay *in vitro*

For osteoclast resorptive function analysis, the osteoclast differentiation of bone marrow macrophages/RAW264.7 cells was conducted in OsteoAssay bone plates (Lonza) at a density of 1 × 10^4 ^cells/well^35^. Either agomir-214-3p (200 μM) or antagomir-214-3p (200 μM) were transfected on day 0 and day 5. Bone slices were ultra-sonicated in 1 mol/L NH4OH to remove adherent cells and stained with 0.1% toluidine blue solution. Bone slice images were captured using electron microscopy. Three fields were randomly selected for each bone slice for further analysis. Pit areas were quantified using Image Pro Plus 6.2 software (Media Cybernetics Inc.).

### Luciferase Assay

*Traf3* mRNA 3′UTR containing the miR-214-3p-binding sequences for the mouse *Traf3* gene was amplified by PCR from mouse genomic DNA. The PCR product was then sub-cloned into the XbaI site downstream of the stop codon in the pGL3 control vector (Promega). The QuickChange site-directed mutagenesis kit (Stratagene) was used to introduce mutations into the seed region of pGL3-*Traf3*, giving pGL3- *Traf3*-mutant, following the manufacturer’s instructions. HEK293 and RAW264.7 cells were seeded in 96-well plates and co-transfected with either 50 nM agomir-NC agomir-214-3p/agomir-214-3p-Mut or antagomir-NC/antagomir-214-3p, 100 ng of luciferase vector (pGL3 constructs) and 25 ng Renilla vector (pRL-TK) using Lipofectamine 2000 (Invitrogen). 24 h after transfection, luciferase activity was measured using the dual-luciferase reporter assay system (Promega). Luminescent signals were quantified by luminometer (Glomax, Promega), and each value from the firefly luciferase construct was normalized by Renilla luciferase assay.

### Bone metastasis analyses for xenograft nude mice

The luciferase-labelled human breast cancer cell line (MDA-MB-231-luc-D3H2LN) was purchased from Caliper Life Sciences Inc., and cultured with DMEM medium (high glucose) with 10% fetal bovine serum (Gibco), 0.1 mM MEM non-essential amino acids and 2 mM L-glutamine. After trypsin digestion, the cell suspension was injected into the left cardiac ventricle of 6-week-old female nude mice at 1 × 10^5^ cells per mouse in 100 μl PBS^19^, which was confirmed by *in vivo* biophotonic imaging immediately after injection (Supplementary Fig. 6a). Bone metastases were monitored and quantified weekly after injection by biophotonic imaging using a Caliper Xenogen Spectrum instrument. At 6 to 8 weeks after xenograft, the mice were sacrificed after biophotonic imaging and the limb bone were collected for microCT scanning and analysis for the the osteolytic metastatic lesions. All experiments were performed in accordance with relevant guidelines and regulations and all experimental procedures were approved by the licensing Committees of Animal Ethics and Experimental Safety of Hong Kong Baptist University.

### Total RNA extraction, reverse transcription and quantitative real-time PCR analysis

Total RNA extraction was extracted using the RNeasy Mini Kit (Qiagen, 74106) according to the manufacturer’s protocol. For mRNA expression, RNA was reverse transcribed into cDNA using a QuantiTect Reverse Transcription Kit according to an established protocol. The primer for mRNAs (*Traf3, Trap, Ctsk* and *Gapdh*) were purchased from Shanghai Integrated Biotech Solutions Co., Ltd. For miRNA expression, the TaqMan primer-probe combinations for miRNA-214-3p and RUN6B were products of Ambion. Real-Time PCR reactions were performed using the StepOnePlus real-time PCR system (Applied Biosystems, Foster City, CA, USA), and the signal was converted into numerical values by StepOne software v2.1 (Applied Biosystems, Foster City, CA, USA). All reactions were performed in triplicate and the cycle threshold (CT) value in each reaction well was recorded. The relative quantification of miR-214 expression was calculated using the 2^−∆∆CT^ method.

### Western blot analysis

Proteins were extracted from the ultracentrifugation pellets and separated on a denatured SDS-polyacrylamide gel before transfer to a polyvinylidene difluoride membrane (0.45 um; Millipore, Bedford, MA, USA). Afterwards, the membranes were blocked with 5% non-fat dry milk in Tris-buffered saline with Tween 20 (TBST) for 1 hour. The membranes were probed with primary anti-TRAF3 (1:200, Santa Cruz, sc-949), anti-PTEN (1:1000, Cell Signaling, #9559), anti-p65 (1:200, Santa Cruz, sc-372), anti-NIK (1:200, Santa Cruz, sc-7211) or anti-NFATc1 (1:1000, Cell Signaling, #8032), respectively. To check the amount of proteins transferred to nitrocellulose membrane, GAPDH was used as control and detected by an anti-GAPDH (1:500; Santa Cruz, sc-25778) antibody. The membranes were then washed three times with TBST and incubated for 1 hour with HRP-conjugated secondary antibodies (1:2000; Santa Cruz, sc-2004). The antigen-antibody complexes were visualized using SuperSignal^®^ West Dura Extended Duration Substrate (Thermo Scientific, Prod # 34075). Supplementary Fig. 7 depicts uncropped western blots.

### Magnetic-activated cell sorting

Bone marrow cells were flushed from the diaphysis of the femur and dissociated to a single-cell suspension by pipetting up and down and passed through 30 μm nylon mesh to remove cell clumps which may clog the column. Cells were centrifuged at 300 g for 10 min and 10^7^ total cells were re-suspended in 200 μl of ice-cold buffer (DPBS without Ca^2+^ and Mg^2+^, with 0.5% bovine serum albumin, and 2 mM EDTA). 10 ug/ml Rat anti-Mouse OSCAR (R&D Systems) was added to the cell suspension and incubated at 4 °C for 1 h. After washing and centrifuge, the cells were re-suspended in 80 μl of ice-cold buffer. 20 μl of Anti-Rat IgG MicroBeads (Miltenyi Biotec) was added per 10^7^ total cells, incubated for 15 min at 4 °C. Cells were washed with 1–2 ml of buffer per 10^7^ cells, centrifuged at 300 g for 5 min, and re-suspended in 500 μl of buffer. A MACS column (Miltenyi Biotec) was placed in the magnetic field of a suitable MACS Separator (Miltenyi Biotec) and prepared by rinsing with 3 ml of buffer. The column was washed by 3 ml of buffer for three times. Flow-through of unlabeled cells (OSCAR^−^ cells) were collected. The column was placed over a collection tube, 5 ml buffer was pipetted onto the column, and the magnetically labeled cells (OSCAR^+^ cells) were flushed out by firmly pushing the plunger into the column. OSCAR^+^ and OSCAR^−^ cells were collected for downstream analysis. To perform phenotypical analysis, the mRNA levels of osteoclast-related marker genes (Rank, Ctsk and Trap5b) in OSCAR+/OSCAR- cells were examined by RT-PCR.

### Micro-CT analyses

Tibiae were collected, and the attached soft tissue was removed thoroughly and fixed in 4% paraformaldehyde. The fixed tibiae were analyzed using micro-CT (vivaCT 40) and software IPL V5.15 at Scanco Medical AG, Swtizerland. Totally 200 slices with a voxel size of 15 μm was chosen at the region of distal femur beginning at the growth plate extending along the tibiae diaphysis to evaluate bone mass, geometry, and trabecular microarchitecture. The whole trabecular bone were isolated for three-dimension reconstruction (Sigma = 1.2, Supports = 2 and Threshold = 180) to calculate the following parameters: bone mineral density (BMD), bone volume/tissue volume (BV/TV), trabecular thickness (Tb.Th), and trabecular number (Tb.N).

### Bone histomorphometric analyses

Tibiae specimens were fixed in 4% PFA for 24 hours and decalcified in fresh 10% EDTA for 4 weeks following paraffin embedding. Thereafter, the trabecular sections at the proximal tibiae and tartrate resistant acid phosphatase (TRAP) staining were performed. Bone static histomorphometric analyses for osteoclast surface (the percent of trabecular bone surface covered by osteoclasts, Oc.S/BS) and osteoclast number per bone perimeter (N.Oc/B.Pm) were made using professional image analysis software (Image J, NIH, USA) under microscope (Leica image analysis system, Q500MC). The bone histomorphometric parameters were calculated and expressed according to standardized nomenclature for bone histomorphometry.

### Synthesis of osteclast-targeting delivery system encapsulating antagomiR-214-3p or *Traf3* 3′UTR plasmid

The antagomir-214-3p, antagomir-NC and plasmid containing *Traf3* 3′UTR or mutant *Traf3* 3′UTR were encapsulated into the osteoclast-targeting delivery system (D-Asp_8_-liposome), respectively, using our previously established protocol^25^. Briefly, the lipids of DOTAP, DOPE, Chol, DSPE-mPEG2000, and DSPE-PEG2000-MAL at a molar ratio of 42:15:38:3:2 dissolved in chloroform were dried into a thin film followed by hydration in PBS. The liposomal solution was then extruded through two membranes (0.2 and 0.1 um) in five times, respectively. Then, the preformed liposome was reacted with D-Asp_8_ peptide with an N-terminal acetylcysteine residue for 2 h at 4 °C. Subsequently, the reacted mixture was purified by Sepharose CL-4B column to ensure removal of the un-conjugated D-Asp_8_ peptide. The liposomal dissolution in 0.5 ml aliquot was mixed with 0.5 ml distilled water containing mannitol and then lyophilized. Finally, the above lyophilized liposome with 15 umol lipids were rehydrated by adding 0.5 ml DNase-Free water containing antagomir-214-3p (750 ug) or *Traf3* 3′UTR plasmid (120 ug) and incubated for 20 min at room temperature. The encapsulation procedure was performed immediately before use and then sterilized by passing through the sterile filter.

### Statistical Analyses

In general, statistical differences among groups were analyzed by one-way analysis of Variance (ANOVA) with a post-hoc test (after normalization to baseline/control group) to determine group differences in the study parameters. Especially, the rescue treatment data were compared by means of two-way ANOVA (genetic background and pharmacological treatment) with full factorial design. When ANOVA revealed differences and when treatment presented homogeneity of variance, we performed Tukey’s HSD test for multiple comparison. For heterogeneous variance, we employed Games-Howell multiple comparison tests. All statistical analyses were performed with SPSS software at version 19.0 (SPSS Inc., Chicago, IL, USA).

## Additional Information

**How to cite this article:** Liu, J. *et al*. Osteoclastic miR-214 targets TRAF3 to contribute to osteolytic bone metastasis of breast cancer. *Sci. Rep.* **7**, 40487; doi: 10.1038/srep40487 (2017).

**Publisher's note:** Springer Nature remains neutral with regard to jurisdictional claims in published maps and institutional affiliations.

## Supplementary Material

Supplementary Information
