# Supplementary material for: Osteoclastic miR-214 targets TRAF3 to contribute to osteolytic bone metastasis of breast cancer
Source: Sci Rep. 2017 Jan 10;7:40487. doi: 10.1038/srep40487 (PMC5223164; doi:10.1038/srep40487)
Supplement: Supplementary Information [file srep40487-s1.pdf]

Osteoclastic miR-214 targets TRAF3 to contribute to osteolytic bone metastasis of breast cancer

Jin Liu[1-3,13], Defang Li[1-4,13], Lei Dang[1-4,13], Chao Liang[1-3,5-7], Baosheng Guo [1,7], Cheng Lu[2,8], Xiaojuan He[1,8], Hilda Y S Cheung [1,5], Bing He[1-3], Biao Liu[1-3,6], Fangfei Li[1-3], Jun Lu[1-3,6], Luyao Wang[1], Atik Badshah Shaikh[1,3], Feng Jiang[1-3], Changwei Lu[1], Songlin Peng[1,9], Zongkang Zhang[10], Bao-Ting Zhang[10], Xiaohua Pan[1,11], Lianbo Xiao[1,12], Aiping Lu[1-8,12,*] & Ge Zhang[1-7,12,*]

Supplementary Figure 1

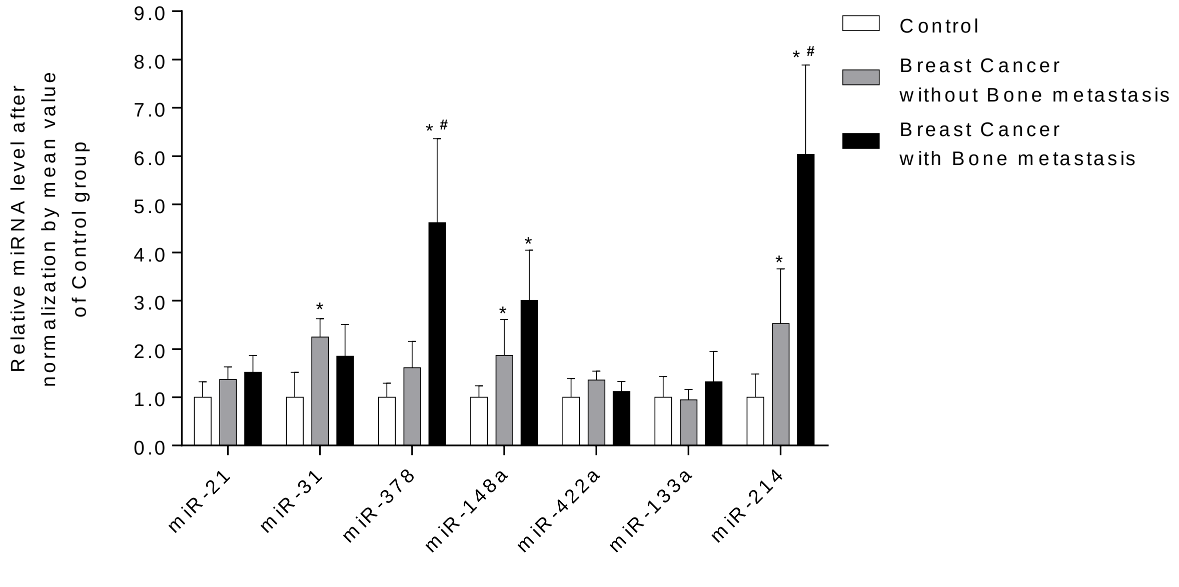

Supplementary Figure 1 Expression profile of osteoclastogenic miRNAs in human bone specimens. The expression of osteoclastogenic miRNAs in bone specimens from breast cancer patient and cancer-free individuals with fracture. Note: The miRNA levels were normalized to U6. *P < 0.05 when compared to the Control group. #P < 0.05 when compared to Breast Cancer without Bone Metastasis group.

Supplementary Figure 2

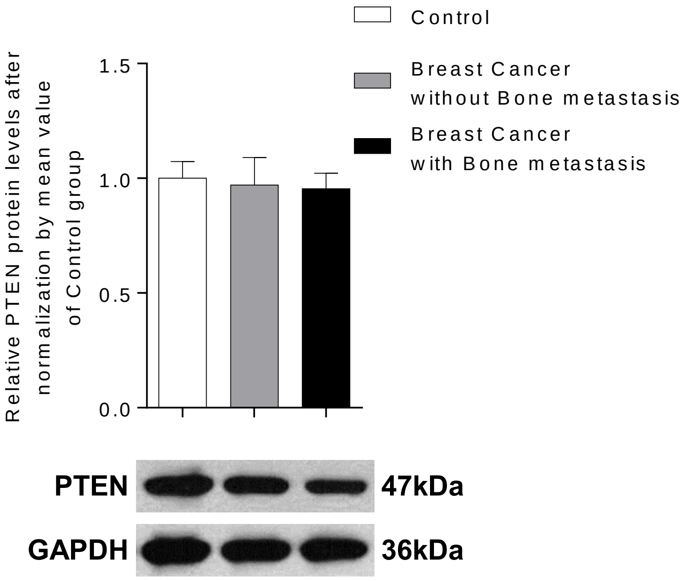

Supplementary Figure 2 The protein expression of PTEN in bone tissues from breast cancer patients. The protein levels of PTEN in bone specimens from breast cancer patient and cancer-free individuals with fracture. Control: cancer-free individuals.

Supplementary Figure 3

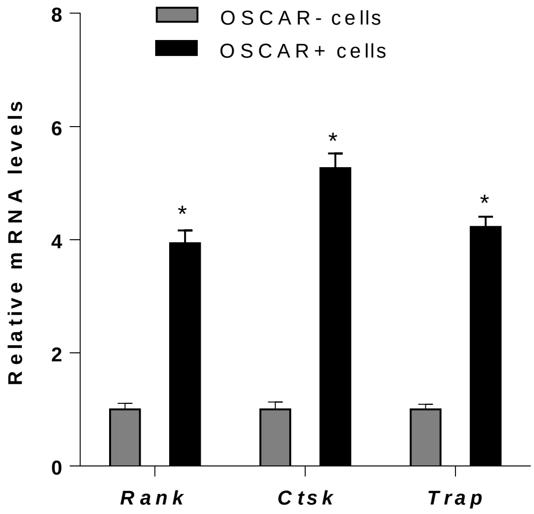

Supplementary Figure 3 Phenotypical analysis of OSCAR$^+$ cells from murine bone marrow. The mRNA levels of *Rank*, *Ctsk* and *Trap5b* in OSCAR$^+$ cells and OSCAR$^-$ cells isolated from bone marrow by MACS. Data are the mean ± s.d. **P* < 0.05.

Supplementary Figure 4

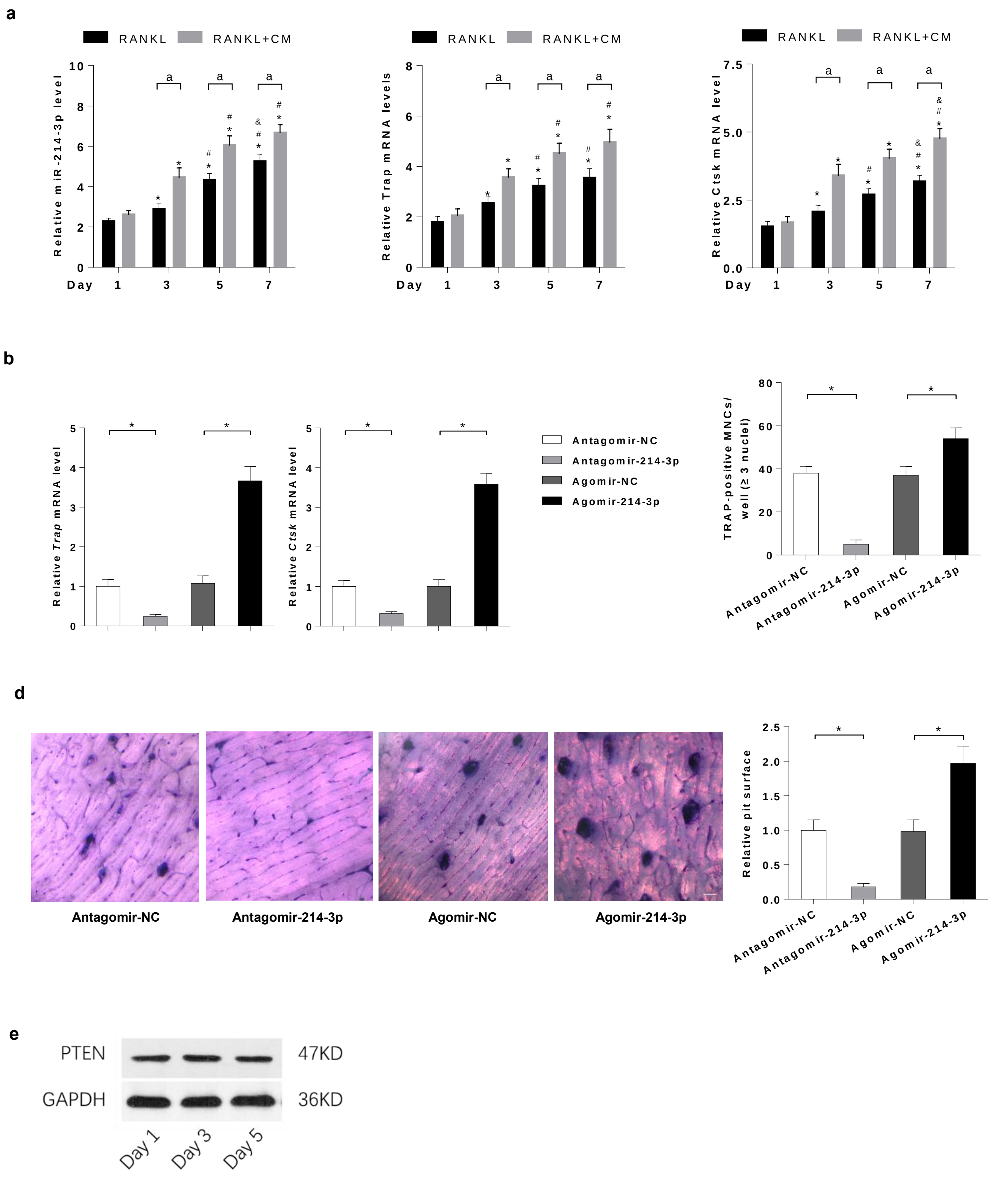

Supplementary Figure 4 miR-214-3p promotes the osteoclast differentiation in the presence of conditioned medium from breast cancer cells *in vitro*. (**a**) Q-PCR analysis of miR-214-3p (left) and osteoclast marker genes (*Trap* and *Ctsk*) (right) in mouse bone marrow macrophage cells (BMMs) at day 1, 3, 5 and 7 after induction by RANKL (5 ng/ml) with or without conditioned medium (CM) from the cultured MDA-MB-231 cells. MiR-214-3p levels were normalized to U6 and osteoclasts marker genes mRNA levels were normalized to *Gapdh*. $^{*}P < 0.05$ compared to the day 1, $^{\#}P < 0.05$ compared to the day 3, $^{\&}P < 0.05$ compared to the day 5. $^{a}P < 0.05$ compared to RANKL group. (**b**) Q-PCR analysis of *Trap* and *Ctsk* mRNA levels in RAW264.7 cells after treatment with antagomir-214-3p or agomir-214-3p or their corresponding negative controls in the presence of RANKL and CM. *Trap* and *Ctsk* mRNA levels were normalized to *Gapdh*. (**c**) The number of TRAP-positive multinucleated cells (MNCs) after treatment with antagomir-

214-3p or agomir-214-3p in the presence of RANKL and CM. (**d**) Representative micrographs of the resorbed bone slices (left) and the areas of bone resorption (right) after treatment with antagomir-214-3p or agomir-214-3p in the presence of RANKL and CM. Scale bar, 100 μm. (**e**) Western blot analysis of PTEN protein expression in RAW264.7 cells during osteoclast differentiation in the presence of RANKL and CM. The data were mean ± s.d. of three experiments in triplicate. **P* < 0.05.

Supplementary Figure 5

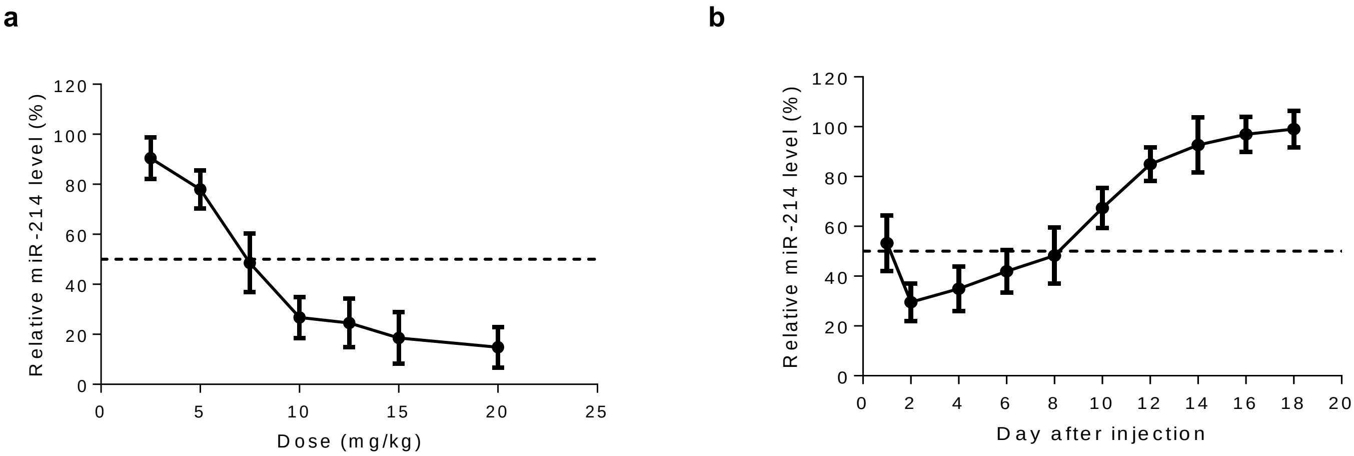

Supplementary Figure 5 Dose-response pattern and persistence of miR-214-3p inhibition of (D-Asp$_8$)-liposome-antagomiR-214-3p in BCX nude mice. (**a**) Dose-dependent miR-214-3p knockdown efficiency in osteoclasts (OSCAR+ cells) was investigated by RT-PCR in combination with magnetic cell sorting (MACS) after tail vein injection of (D-Asp$_8$)-liposome-antagomiR-214-3p with the antagomir-214-3p dose ranged from 2.5 mg/kg to 20 mg/kg. (**b**) After a single injection of (D-Asp$_8$)-liposome-antagomir-214-3p with antagomir-214-3p dose at 10 mg/kg, persistence of miR-214-3p knockdown in osteoclasts (OSCAR+ cells) was investigated by RT-PCR in combination with LCM. Data were mean ± s.d. n=5~6 per group.

Supplementary Figure 6

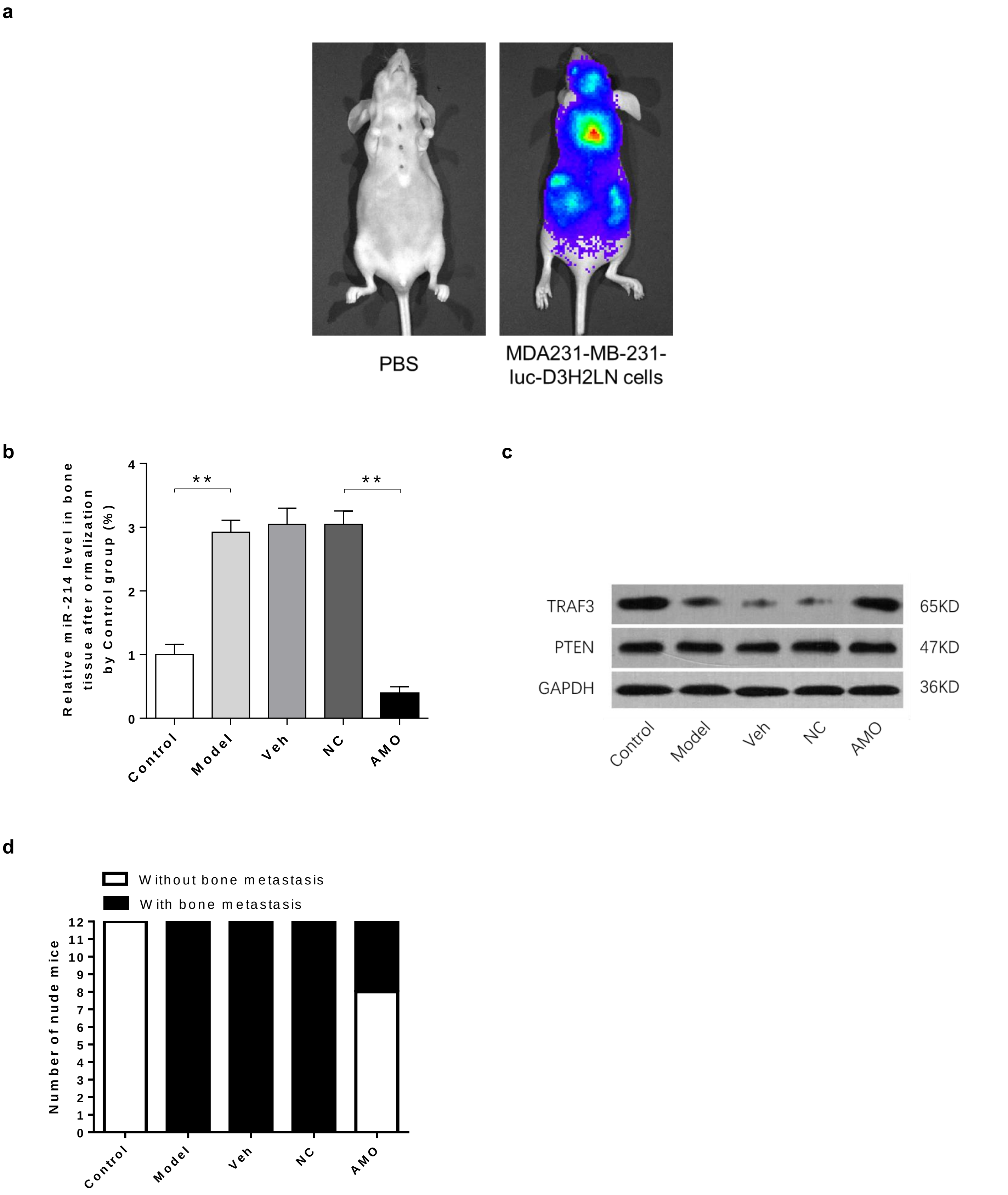

Supplementary Figure 6 The effects of osteoclast-targeted miR-214-3p inhibition on osteolytic bone metastasis in nude mice with breast cancer xenografts. (a) Biophotonic images showing bioluminescence signal in the nude mice after left ventricular injection with MDA231-MB-231-luc-D3H2LN cells. No signal was detected in the

mice injected with PBS. **(b)** Q-PCR analysis of miR-214-3p levels in bone tissue from the indicated group after treatment. n=8 for each group. **(c)** Western blot analysis of TRAF3, PTEN and GAPDH levels in bone tissue from the indicated group after treatment. **(d)** The number of nude mice with/without bone metastasis in the long bone (forelimb, hindlimb) and spine area detected by bioluminescence imaging. Note: $^{**}P < 0.01$. One-way analysis of variance (ANOVA) with a post-hoc test was performed. Control: nude mice without breast cancer xenografts, Model: nude mice with breast cancer xenografts and administrated with PBS alone, Veh: nude mice with breast cancer xenografts and administrated with $(D\text{-}Asp)_8$-liposome alone, NC: nude mice with breast cancer xenografts and administrated with $(D\text{-}Asp)_8$-liposome-antagomir negative control, AMO: nude mice with breast cancer xenografts and administrated with $(D\text{-}Asp_8)$-liposome-antagomir-214-3p.

Supplementary Figure 7

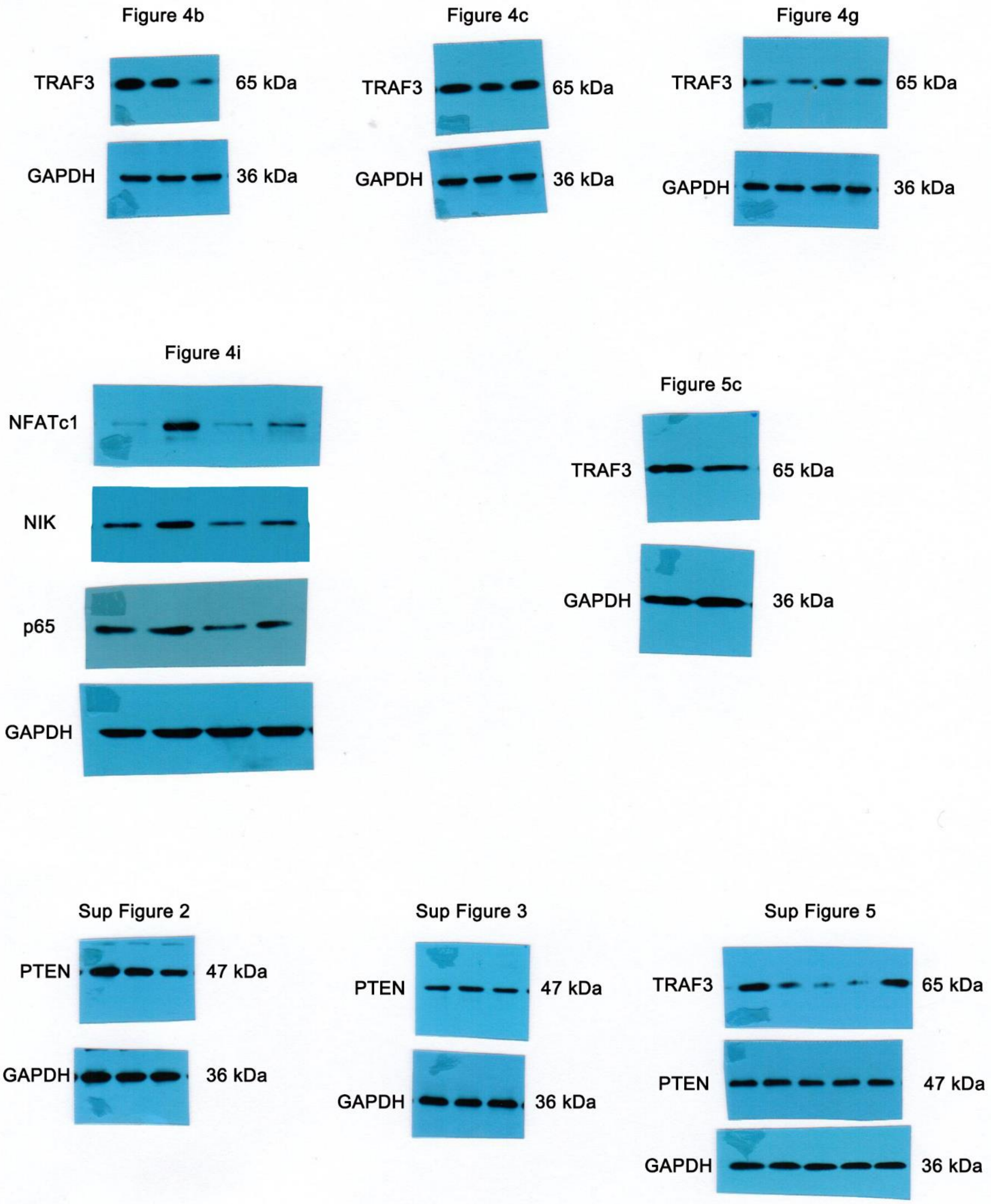

Supplementary Figure 7 The uncropped western blot scans.

Supplementary Table 1 Clinical features of the bone specimen donors

Groups	Age	T score for BMD at Lumbar Spine	Serum BGP (ng/mL)	Serum ALP (U/L)	Serum TRAP-5b (U/L)	Serum BSP (ng/mL)
CON (without cancer)	52±6	-1.89±0.43	5.81±0.53	74.4±7.1	5.97±0.62	36.1±5.3
BC	55±7	-1.95±0.39	5.94±0.51	73.8±7.4	6.36±0.64¶	45.6±4.7
OBM	53±10	-2.29±0.31§¶	6.14±0.54	79.6±7.8	7.37±0.65§¶	67.2±5.9§¶

CON (without cancer): control patients without cancer

BC: breast cancer patients without osteolytic bone metastasis

OBM: breast cancer patients with osteolytic bone metastasis

§ $P<0.05$ as compared to PM or BC group

¶ $P<0.05$ as compared to CON group

Supplementary Table 2 The biochemistry parameters

Groups	**ALT** (U/L)	**AST** (U/L)	**BUN** (mg/dL)
**BCX+PBS**	66.5±10.4	148.3±7.9	16.5±4.9
**BCX+Veh**	64.6±9.5	146.5±9.4	15.6±4.3
**BCX+NC**	65.3±12.5	142.7±17.1	15.9±3.7
**BCX+AMO**	65.8±13.3	139.3±15.4	17.1±5.4

ALT: alanine aminotransferase; AST: aspartate aminotransferase; BUN: blood urea nitrogen. Data were means ± sd. n=5~7 per group.
